# Alterations in Oral Microbiota of Differentiated Thyroid Carcinoma Patients With Xerostomia After Radioiodine Therapy

**DOI:** 10.3389/fendo.2022.895970

**Published:** 2022-08-26

**Authors:** Baiqiang Lin, Fuya Zhao, Yang Liu, Jiayu Sun, Jing Feng, Lei Zhao, Haoran Wang, Hongye Chen, Wei Yan, Xiao Guo, Shang Shi, Zhiyong Li, Shuang Wang, Yu Lu, Jianjun Zheng, Yunwei Wei

**Affiliations:** ^1^ Pancreatic and Gastrointestinal Surgery Division, HwaMei Hospital, University of Chinese Academy of Sciences, Ningbo, China; ^2^ Ningbo Clinical Research Center for Digestive System Tumors, Ningbo, China; ^3^ Oncology and Laparoscopy Surgery, The First Affiliated Hospital of Harbin Medical University, Harbin, China; ^4^ Imaging Center, HwaMei Hospital, University of Chinese Academy of Sciences, Ningbo, China; ^5^ Ningbo Clinical Medical Research Center of Imaging Medicine, Ningbo, China; ^6^ Gastrointestinal Surgery, The First Affiliated Hospital of Fujian Medical University, Fuzhou, China; ^7^ General Surgery, Zhujiang Hospital, SouthernMedical University, Guangzhou, China

**Keywords:** oral microbiota, xerostomia, differentiated thyroid carcinoma, radioiodine therapy, 16S rRNA

## Abstract

**Background and Aims:**

Oral xerostomia remains one of the most common complications of differentiated thyroid carcinoma patients (DTC) after radioiodine therapy (RAI). Environmental factors in the etiology of xerostomia are largely unknown. We aimed to characterize the oral microbiota signatures and related biological functions associated with xerostomia and identify environmental factors affecting them.

**Methods:**

Saliva was collected from 30 DTC patients with xerostomia (XAs), 32 patients without xerostomia (indicated as non-XAs) following RAI after total thyroidectomy, and 40 healthy people (HCs) for 16S rRNA sequencing analysis.

**Results:**

The oral microbiota of XAs and non-XAs exhibited significant differences in α and β diversities and bacterial taxa. The abundance of *porphyromonas*, *fusobacterium*, and *treponema_2* were significantly higher in XAs, while the abundance of the *streptococcus* was lower in the microbiota of non-XAs. *Fusobacterium*, and *porphyromonas* were negatively correlated with unstimulated/stimulated whole salivary secretion (USW)/(SWS), while *fusobacterium*, *porphyromonas*, and *treponema_2* genera levels were positively associated with cumulative radioiodine dose. PICRUSt2 and BugBase suggested a significant difference in the expression of potentially_pathogenic, anaerobic, gram_negative, the arachidonic acid metabolism, and lipopolysaccharide (LPS) biosynthesis between XAs and non-XAs, possibly interdependent on radioiodine-induced inflammation. NetShift analysis revealed that *porphyromonas* genus might play as a key driver during the process of xerostomia. Five genera effectively distinguished XAs from non-XAs (AUC = 0.87).

**Conclusion:**

Our study suggests for the first time that DTC patients with xerostomia after RAI display microbiota profiles and associated functional changes that may promote a pro-inflammatory environment. Dysbiosis of the oral microbiota may contribute to exacerbating the severity of xerostomia. Our results provide a research direction of the interaction mechanism between oral microbiota alteration and the progress of xerostomia.

## Introduction

Sialadenitis is a relatively common complication of radioactive iodine therapy (RAI) in DTC patients (1), which may result in subsequent xerostomia (2). Research reported the incidence of salivary gland dysfunction to be as high as 72.73% (3). Xerostomia manifests abnormal saliva excretion, adversely affecting patients’ life quality in the long term. Saliva is essential for oral homeostasis, and its reduction increases the risk of oral morbidity. Prolonged saliva reduction leads to dental caries, dysphagia, and multiple psychological effects, including depression and anxiety, which are particularly pronounced in female patients (4–6). Several methods have been used to limit this complication, including the use of amifostine, chewing gum, acidic food, and Cholinergic drugs (7–10). The effect of sialogogues like sour liquids on RAI-induced xerostomia is controversial (11). To date, no interventions have been shown to be uniformly effective in treating and preventing xerostomia. Innovative preventive measures and strategies to identify those with RAI at risk of xerostomia progression are urgently needed.

Xerostomia following RAI was shown to be dose-related with a dose-response relationship between symptoms of salivary morbidity and activity (>100 mCi; 3.7 GBq) (12). The concentration of iodine in the salivary glands is 30 to 40 times that of the blood. After the therapeutic dose, the salivary gland dose could be as high as 6 Gy (13). The inflammatory scarring of salivary ducts and loss of fluid-producing acinar cells caused by radiation can promote the occurrence of xerostomia (14). There is still a huge gap in knowledge on environmental factors involved in the etiology of xerostomia. Studies have shown that apoptosis of salivary epithelial cells and inflammation of salivary tissue induced by RAI impaired epithelial barrier integrity (14), resulting in bacterial colonization. Once bacteria have invaded the ductal epithelial or acinar cells (15), they may release bacterial products that bind to salivary gland cell Toll-like receptor (TLR) to release inflammatory cytokines, which further aggravate salivary gland inflammation. Studies have shown that oral bacteria *in vitro* can cause salivary acinar cell apoptosis (16). Yet, although a large number of studies have shown that the oral microbiota can affect the progression of primary Sjögren’s syndrome, and salivary secretion has a stronger influence on the oral microbiota (17, 18). To date, no study has shown whether the oral microbiota of patients with xerostomia after RAI is altered.

Hence, this study sampled patients’ saliva and performed 16S rRNA gene sequencing. We aimed to identify alterations in the oral microbiota associated with DTC patients with xerostomia after RAI. We also aimed to identify functional changes and contributing factors of these microbiota alterations.

## Methods

### Ethics

The Ethics Committee of the First Affiliated Hospital of Harbin Medical University approved all protocols applied in this study (201816). All volunteers signed written informed consent for participation in the study (NCT03574051). Written informed consent was obtained from those who agreed to participate.

### Study Design

In a cross-sectional study, DTC patients between 18 and 65 years who had undergone total thyroidectomy and RAI treatment between 2015 and 2017 were enrolled in our study (**Figure S1**). Only patients from 19 to 41 months from RAI treatment end were included, with a median posttreatment time of 32 months. All the patients were taking suppressive doses of levothyroxine and had normal thyroid hormone levels. Patients were excluded if they were using corticosteroids at the time of RAI treatment. Patients who were receiving a drug that could affect salivary gland function were also excluded. Three hundred four patients were telephonically interviewed, of which 82 reported dry mouth symptoms. Two hundred twenty-two reported no dry mouth symptoms. Patients reporting dry mouth symptoms were followed up for evaluation of their salivary flow rate and considered xerostomic when the unstimulated total salivary flow was less than or equal to 0.2 mL/min and/or the stimulated whole saliva flow of less or equal to 0.7 mL/min (19, 20). Based on these criteria, 30 patients were diagnosed as xerostomic (the XA group). Subsequently, 32 patients without dry-mouth symptoms with matching age, gender, and BMI were randomly selected (non-XA group) out of 222 patients without dry-mouth symptoms. At the same period, 40 sex, body mass index (BMI), and age-matched healthy people (HCs)(the HC group) were recruited from the Health Screening Center. All participants apply the same exclusion criteria (**Supplementary Material**).

### Sample Collection

The participants were given oral hygiene instruction and oral health examination before follow-up. Sample collection was performed at a median follow-up of 32 months after RAI treatment. Plasma and saliva samples were obtained from each subject and healthy people. Sampling and storage methods of saliva and plasma samples and analysis of clinical parameters were detailed in **Supplementary Material**. Plasma samples from participants were used for the detection of thyroid function, thyroglobulin, liver function, and blood lipid levels. Saliva samples were used for the 16S rRNA gene sequence.

### Unstimulated Whole Saliva Secretion and Stimulated Whole Saliva Secretion

Unstimulated whole saliva secretion (UWS) was collected using the drooling method, i.e., within 5 minutes, instruct the participants to spit the saliva accumulated at the bottom of the mouth into a sterile graduated test tube along a sterile funnel. Stimulated whole saliva secretion (SWS) was collected by dripping 2% citric acid on the front of the subject’s tongue.

### Xerostomia Inventory Score

The severity of post-RAI xerostomia in DTC patients was assessed by the Xerostomia Inventory score (XI Score), the applicability of which has been validated in other studies (19, 21). The English version of the XI Score was translated into a Chinese version for participants to conduct the questionnaire. In this study, all participants in the XA group, the non-XA group, and the HC group were administered questionnaires. The XI Score includes 11 options, each of which is quantified with a Likert scale totaling 5 points. Participants were asked to choose one of five responses to each option (score one-never; score two-rarely; score three-occasionally; score four-frequently; and score five-very often). The XI Score’s 11 options have a total score between 11 and 55 points. According to the size of the participants’ XI total score, the participants were divided into three grades (11-23 points: no to mild xerostomia; 24-39 points: moderate to severe xerostomia; 40-55 points: severe to extreme xerostomia).

### gDNA Extraction and 16S rRNA Gene Sequencing

The methods for gDNA extraction and 16S rRNA gene sequencing were as described in our previous study (22). Bacterial genomic DNA isolation was performed on all saliva samples using E.Z.N.A.^®^ soil DNA kit (Omega Bio-tek, Norcross, GA, U.S.). Then the extraction quality, concentration, and purity of DNA were tested. PCR amplification experiments were performed on the V3-V4 region of the microbial 16S rRNA gene, and the PCR product was recovered, purified, and quantified. Library construction was performed using the NEXTFLEX Rapid DNA-Seq Kit. Sequencing analysis was performed using Illumina Miseq high-throughput sequencing platform (22). The raw sequence data of this study were stored in the Sequence Read Archive (SRA) database of NCBI. The accession number of the database is PRJNA 784772.

### Bioinformatics and Statistical Analysis

The α and β diversity of microbial communities was calculated by Quantitative Insights Into Microbial Ecology (QIIME, Version 1.9.1), and the results were displayed by R software (Version 4.1.0) (22). Differences were analyzed at the genus and species level using the Wilcoxon rank-sum test. The genera with a prevalence greater than 50% and maximum proportions greater than 0.001 were analyzed for differences and only genera with P < 0.05 were considered statistically significant. Predicted functional composition maps were annotated into the KEGG pathway (level 3) based on 16S rRNA-seq sequences using PICRUSt2. BugBase was used for oral microbial phenotype prediction. The phenotype types include Pathogenic, Mobile Element Containing, Oxidative Stress Tolerant, Gram-Negative, Gram-Positive, Biofilm Forming, and Oxygen Utilizing (23). Correlations between variables (genera and clinical parameters) were calculated using Spearman’s correlation, and the correlation (Student’s t-test, |correlation coefficient (R)| > 0.3, P < 0.05) are presented using a network diagram or a heatmap (Cytoscape, Version 3.2.1). RDA analysis is a redundancy analysis, which can reflect microbial samples and environmental factors on the same two-dimensional ranking map. Fitting variables to the RDA plot using the “envfit” function from the Vegan package and using linear constraints. Linear discriminant analysis (LDA) effect size (LEfSe) analysis was used to assess differences in oral bacterial genera (P < 0.05 and Log10 LDA > 3).

Significantly positive pairwise correlations were established by CCREPE (Compositionality Corrected by REnormalization and PErmutation) software package. Applying NetShift analysis based on correlation coefficients to build Driver and core hub genera (24). Microbial drivers were shown by calculating the Neighbor Shift (NESH) score and node size. The threshold set for the correlation coefficient was 0.5. Analysis of clinical parameters was performed by the Statistical Package for the Social Sciences (SPSS) version 25.0.

## Results

### Patients’ Clinical Characteristics and 16S rRNA Sequencing

A total number of 62 DTC patients (the XA group, n=32/non-XA group, n=30) and 40 HCs were included in the final cohort (**Figure S1**). The patients’ clinical characteristics and oral health status were compared between the three groups. Generally, cumulative radioiodine dose (P < 0.001), USW (P < 0.001), SWS (P < 0.001), XI-score (P < 0.001) and OHI (P < 0.006) were higher in XA group compared with non-XA and HC groups (**Table 1**). We filtered the reads obtained by sequencing and obtained a total of 7,735,905 sequence reads (102 samples), with an average number of 75,842 sequences (the minimum of 48,806; the maximum of 89,426). Rarefaction curves based on Ace and Shannon diversity indicated sufficient sequencing depth (**Figures S2 A, B**). Pan/core species curves also confirmed the above results (**Figures S2C, D**). There were 4120 operational taxonomic units (OTUs), 1914 species, 956 genera, 404 families, 190 orders, 87 classes, and 39 phyla annotated to the Silva database for 102 samples.

**Table 1:** Clinical and demographic features.

**Characteristics**	**XA group** **(n = 30)**	**non-XA group** **(n = 32)**	**HC group** **(n = 40)**	**P1 value** **(XA vs.non-XA)**	**P2 value** **(XA vs. HC)**	**P3 value** **(non-XA vs. HC)**
Sex (M/F) #	7/23	8/24	10/30	0.878	0.872	1.000
Age (years, mean ± SD) *	43.20 ± 6.81	43.19 ± 8.46	40.05 ± 9.44	0.899	0.097	0.143
BMI (kg/m^2^, mean ± SD) *	23.09 ± 1.88	23.12 ± 1.52	23.72 ± 2.05	0.597	0.687	0.359
cumulative radioiodine dose (mCi, mean ± SD) §	253 ± 72	166 ± 89	NA	0.001*	NA	NA
OHI (median, interquartile) §	2, (1-2)	1, (1-2)	1, (1-2)	0.006*	0.002*	0.803
Current smoking status (n, %) #	5, (16.7)	7, (21.9)	5, (12.5)	0.604	0.622	0.289
Current drinking status (n, %) #	4, (13.3)	5, (15.6)	6, (15.0)	0.798	0.844	0.942
Salivary gland function						
SWS§ (ml/min, mean ± SD) §	0.30 ± 0.09	1.01 ± 0.15	1.17 ± 0.15	0.001*	0.001*	0.001*
USW§ (ml/min, mean ± SD) §	0.09 ± 0.02	0.37 ± 0.06	0.53 ± 0.13	0.001*	0.001*	0.001*
Scale-XI Score§ (mean ± SD) §	41 ± 3	18 ± 4	16 ± 4	0.001*	0.001*	0.07
Tumor stage#						
T1-2/T3-4	9/21	15/17	NA	0.173		
Node stage #						
N0/N1	0/30	0/32	NA	NA		
M stage #						
M0/M1	30/0	32/0	NA	NA		
Thyroid function						
fT3 (pg/mL, mean ± SD) §	2.56 ± 0.30	2.51 ± 0.32	2.77 ± 0.26	0.545	0.004*	0.001*
fT4 (ng/dL, mean ± SD) §	1.29 ± 0.29	1.22 ± 0.16	1.06 ± 0.09	0.41	0.01*	0.001*
TSH (µIU/mL, mean ± SD) §	1.63 ± 1.62	1.71 ± 2.18	2.27 ± 1.19	0.91	0.014*	0.001*
Tg (IU/mL, mean ± SD) §	0.34 ± 0.64	0.40 ± 1.07	5.90 ± 3.68	0.783	0.001*	0.001*
Hepatic function						
ALT (U/L, mean ± SD) §	15.51 ± 5.10	16.33 ± 4.71	15.20 ± 7.12	0.278	0.458	0.088
AST (U/L, mean ± SD) §	19.73 ± 4.38	20.75 ± 5.30	18.47 ± 4.00	0.549	0.313	0.077
ALB (g/L, mean ± SD) §	44.16 ± 3.42	43.10 ± 9.35	45.17 ± 2.67	0.39	0.352	0.982
TP (g/L, mean ± SD) §	75.78 ± 3.42	74.13 ± 3.80	74.07 ± 3.69	0.477	0.068	0.362
GLB (g/L, mean ± SD) §	29.57 ± 2.47	29.58 ± 2.98	28.83 ± 3.39	0.126	0.339	0.461
TBIL (µmol/L, mean ± SD) §	13.10 ± 4.55	11.89 ± 2.89	12.29 ± 3.34	0.698	0.877	0.717
DBIL (µmol/L, mean ± SD) §	2.38 ± 0.84	2.25 ± 0.83	2.21 ± 0.61	0.667	0.656	0.781
IBIL (µmol/L, mean ± SD) §	10.63 ± 3.99	9.70 ± 2.32	10.19 ± 3.03	0.893	0.924	0.713
GGT (µmol/L, mean ± SD) §	23.28 ± 10.39	22.68 ± 11.67	22.94 ± 14.10	0.673	0.342	0.38
AKP (µmol/L, mean ± SD) §	83.58 ± 20.53	81.68 ± 29.80	80.77 ± 22.30	0.281	0.79	0.593
Plasma lipid						
CHOL (µmol/L, mean ± SD) §	4.72 ± 0.76	4.89 ± 1.02	4.66 ± 0.47	0.617	0.976	0.544
TG (µmol/L, mean ± SD) §	1.49 ± 0.78	1.19 ± 0.61	1.26 ± 0.39	0.098	0.288	0.255
HDL (µmol/L, mean ± SD) §	1.22 ± 0.21	1.20 ± 0.17	1.17 ± 0.10	0.652	0.302	0.59
LDL (µmol/L, mean ± SD) §	3.28 ± 0.45	3.22 ± 0.75	3.33 ± 0.30	0.683	0.687	0.405
VLDL (µmol/L, mean ± SD) §	0.25 ± 0.11	0.24 ± 0.12	0.26 ± 0.25	0.455	0.079	0.587
APOA (µmol/L, mean ± SD) §	1.47 ± 0.17	1.45 ± 0.19	1.45 ± 0.07	0.725	0.416	0.847
APOB (µmol/L, mean ± SD) §	0.90 ± 0.12	0.90 ± 0.22	0.92 ± 0.06	0.827	0.525	0.816
Lpa (µmol/L, mean ± SD) §	124.99 ± 80.68	143.29 ± 141.25	118.80 ± 70.18	0.938	0.877	0.986

*Student’s t-test; ^§^Mann-Whitney U test; ^#^Chi-Square test. BMI, OHI, oral hygiene index; body mass index. Measurement data are expressed as the mean ± SD. F/M, female/male; BMI, body mass index; OHI, oral hygiene index; SWS, stimulated whole saliva; USW, unstimulated whole saliva; XI score, Xerostomia inventory; TSH, thyroid-stimulating hormone; fT4, free thyroxine; fT3, free triiodothyronine; AST, aspartate transaminase; ALT, alanine aminotransferase; TP, total protein; GLB, globulin; TBIL, total bilirubin; DBIL, direct bilirubin; IBIL, indirect bilirubin; ALB, album; GGT, gamma-glutamyl transpeptidase; AKP, alkaline phosphatase; LDH, lactate dehydrogenase; BUN, blood urea nitrogen; Cr, creatinine; UA, uric acid; GLU, glucose; CHOL, total cholesterol; TG, triacylglycerol; LDL, low-density lipoprotein; HDL, high-density lipoprotein; VLDL, very-low-density lipoprotein; ApoA, apolipoprotein A; ApoB, apolipoprotein B; Lpa, lipoprotein(a); Tg, Thyroglobulin; and SD, standard deviation. *P-value < 0.05. NA, Not Applicable.

### Variations in the Oral Microbiota Diversity in XAs, Non-XAs, and HCs

**Figure 1:**
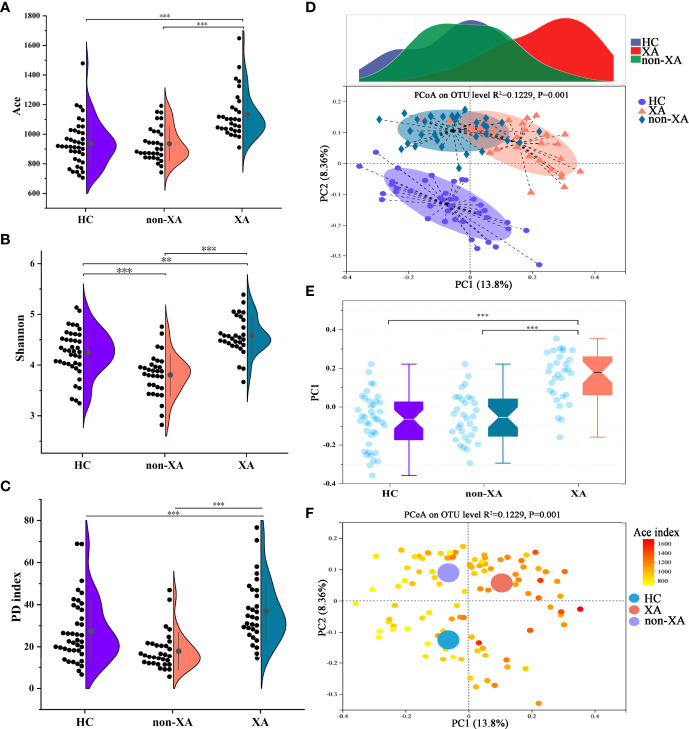
Oral microbial characterization among XAs, non-XAs, and HCs. The α diversity index of Ace **(A)**, Shannon **(B)**, and PD index **(C)** in XAs was significantly increased compared to non-XAs and HCs. **(D, E)** The principal coordinate analysis (PCoA) revealed that the β-diversity for the oral microbiota of XAs was also clearly separated from non-XAs and HCs. **(F)** PCoA as in **(D)**, colored according to Ace index. XAs, xerostomia patients; non-XAs, patients without xerostomia; HCs, healthy controls; PD, phylogenetic diversity, OTUs, operational taxonomic units.

The microbial α-diversity of XAs was compared with non-XAs and HCs based on Ace, Shannon, phylogenetic diversity (PD), Simpson, Sobs, and Chao indexes (**Figures 1A–C** and **S2A–C**). As shown in **Figures 1** and **S2**, the oral microbiota of XAs had a statistically higher α-diversity than non-XAs and HCs (Wilcoxon rank-sum test/P-value < 0.001). The richness and diversity (Simpson and Shannon) of the oral microbiota of non-XAs were statistically higher than HCs (Wilcoxon rank-sum test/P-value < 0.01, **Figures 1B** and **S3A**).

Differences in β-diversity at the OTU level were assessed using PCoAs and Adonis tests (with 999 permutations) based on the Unweighted-UniFrac distance metrics. Consistent with the trend of differences in α diversity among groups, the β-diversity for the oral microbiota of XAs was also clearly separated from non-XAs and HCs (**Figures 1D, E**, and **S3D**). Furthermore, Non-metric multidimensional scaling analysis (NMDS) based on the Unweighted-UniFrac distance also confirmed the results mentioned above (**Figures S3E–G**). With the increase of α-diversity, the samples among three groups with regard to β-diversity showed a clear differentiation gradient (**Figures 1F** and **S3H**). These results collectively suggested that the oral microbiota composition in XAs significantly differed from non-XAs and HCs.

### Microbiota Composition and Comparison Between XAs, Non-XAs, and HCs

**Figure 2:**
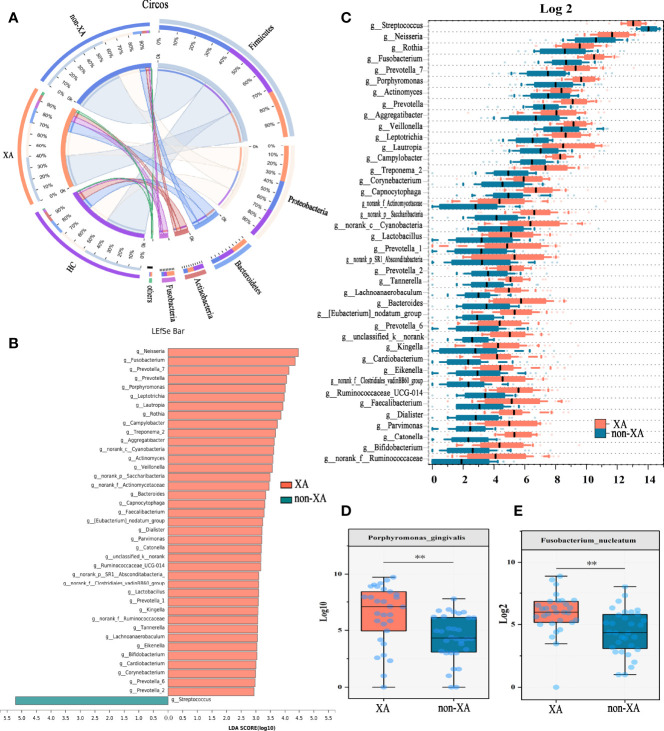
Oral microbial phylotype variation at phylum, genus, and species levels. **(A)** The Circos plot was applied to demonstrate differences in phylum-level microbial composition among the three groups. The inner-circle on the left represented the microbial structural composition at the phylum level of each group. The outer circle on the left represented different groups. The outer circle on the right represented the percentage of phylum in different groups. The width of the bands represented the proportion or relative abundance of the phylum. **(B)** Species with the most significant differences between XAs and non-XAs were identified using LEfSe analysis based on the non-parametric factorial Kruskal-Wallis sum-rank test. XAs-enriched genera were shown in pink and non-XAs-enriched genera were shown in light blue. Genera with LDA values greater than three were reserved. **(C)** At the genus level, genera significantly different between XAs, and non-XAs were compared by Mann-Whitney U-tests (P < 0.05). At the species level, *porphyromonas gingivalis* **(D)** and *fusobacterium nucleatum* **(E)** were enriched in XAs compared to non-XAs. LEfSe, Linear discriminant analysis Effect Size (LEfSe); LDA, linear discriminant analysis; XAs, Xerostomia patients; non-XAs, patients without xerostomia; HCs, healthy controls.

In addition, the oral microbiota structure was analyzed at the phylum level. The average overall bacterial composition in XAs differed from non-XAs and HCs. The proportions of Proteobacteria (31% vs. 26%, 26%), Bacteroidetes (14% vs. 7%, 13%), and Fusobacteria (7% vs. 2%, 3%) were higher, while those of Firmicutes (40% vs. 60%, 49%) were lower in the XA group than in the non-XA and HC group (**Figures 2A** and **S4A**). Moreover, the Firmicutes/Bacteroidetes (F/B) ratio in the former was significantly lower than that in the latter (Wilcoxon rank-sum test, P < 0.01, P < 0.05, respectively; **Figure S4D**). The microbiota composition between XAs, non-XAs, and HCs differed at family and genera levels (**Figures S4B,C**).

The twenty most abundant microbial taxa in each group were shown in **Figure S4C**. To find the specific communities associated with xerostomia, the composition of oral microbiota in XAs and non-XAs was compared using LEfSe analysis. We applied two different analysis methods to explore changes in the saliva microbiota. At the genus levels, a total of 40 discriminative features were found by LEfSe analysis (LDA value > 3, P-value < 0.05, **Figure 2B**). In addition, we also applied the Mann-Whitney U test to identify differential bacterial genera. Genera with maximum proportions > 0.001 and a prevalence > 50% were retained, and 40 differential genera were identified in this analysis. Amazingly, the differential genera identified by the two different analysis methods described above were completely consistent, revealing the stability of saliva microbiota profiling data. The relative abundance of *neisseria*, *fusobacterium*, *porphyromonas*, *veillonella*, *prevotella*, *corynebacterium*, *capnocytophaga*, etc., was increased in the saliva of XAs compared to non-XAs at P-value < 0.05 (**Figure 2C**). In contrast, the abundance of *streptococcus* was decreased in XAs samples compared to non-XAs (P < 0.05, **Figure 2C**). At the species level, the abundance of *porphyromonas gingivalis* and *fusobacterium nucleatum* was enriched in XAs compared non-XAs (P < 0.05, **Figures 2D, E**).

**Figure 3:**
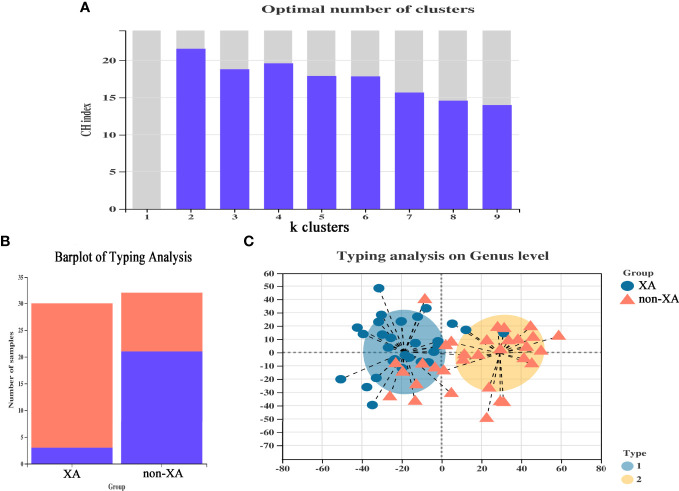
Enterotype analysis of xerostomia patients. **(A)** CH index map, selected the number of clusters when the CH index reaches the maximum to classify the oral microbiota, and optimal classification is achieved when divided into two community types. **(B)** The classification of each group of samples constitutes a histogram, and different colors represented different classifications. **(C)** The plot of PCoA of salivary samples using partitioning around medoids. PCoA, principal coordinate analysis.

To further differentiate the characteristics of the oral microbiota of non-XAs from XAs, we clustered the oral microbiota into different oral types based on the relative abundance of salivary genera which distinguish non-XAs from XAs. The oral microbiota could be clustered into two distinct types (**Figure 3A**). Type I oral type is characterized by a higher relative abundance of *streptococcus*, *neisseria*, *haemophilus* and *fusobacterium*. In contrast, an overabundance of *streptococcus*, *haemophilus*, *neisseria*, and *gemella* in the saliva is characteristic of type II oral type. A large percentage of XAs (27/30) were observed to have the type I oral type, while non-XAs (21/32) had the type II oral type (**Figure 3B**). PCoA revealed both XAs and non-XAs in each community type (**Figure 3C**).

### Association of Oral Microbiota With Clinical Parameters

**Figure 4:**
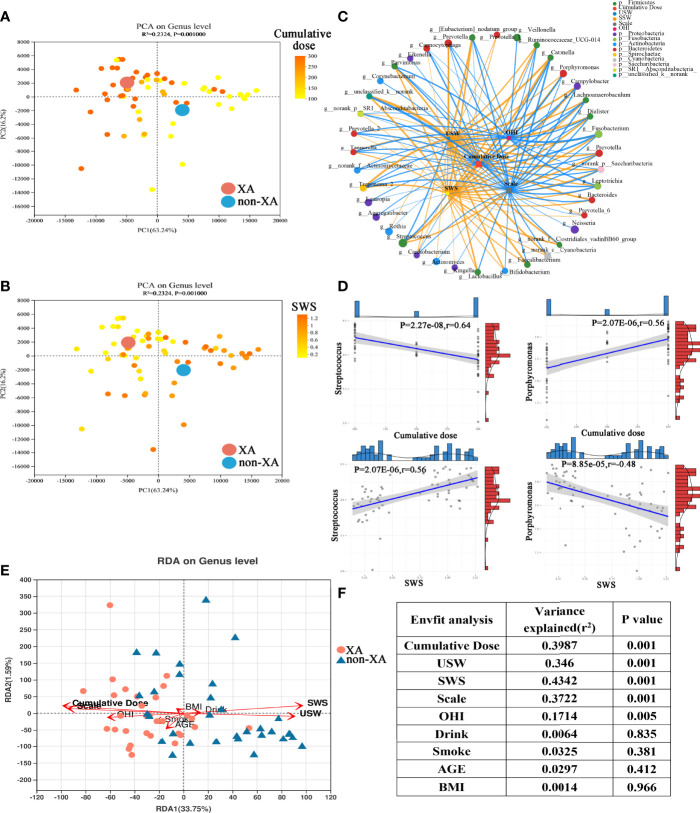
Association of oral microbiota with clinical parameters. PCoA as in (**Figure S3D**), colored according to cumulative radioiodine dose (mCi) **(A)** and stimulated whole salivary secretion (SWS, mL/min) **(B)**. **(C)** Spearman’s correlation between the top 40 abundant oral microbial genera and clinical parameters was shown through a co-correlation network. The width of the line represented the magnitude of correlations, orange indicated a positive correlation, and light blue indicated a negative correlation; the size of the nodes indicated the relative abundance of the genus, and the correlation threshold was set to 0.3. **(D)** Linear correlation analysis revealed a significant correlation between *streptococcus*, *porphyromonas*, and cumulative radioiodine dose, SWS in XAs and non-XAs. **(E)** Triplots of redundancy analysis. Explanatory variables were shown in orange. **(F)** ANOVA-like significance test of the explanatory variables. PCoA, Principal coordinate analysis; XAs, xerostomia patients; non-XAs, patients without xerostomia.

According to the PCA diagram colored on the ground of cumulative radioiodine dose and stimulated whole oral secretion (SWS, mL/min), we find that cumulative radioiodine dose and SWS result in a clear differentiation gradient among the samples with regard to β-diversity (**Figures 4A, B**). We used Spearman’s correlation analysis to explore the correlation of saliva microbiota with patient clinical parameters. The saliva microbiota included 40 top abundance microbial genera identified above. And the clinical parameters were saliva secretion index (SSW, USW, XI score), cumulative radioiodine dose, oral hygiene index (OHI), smoking and drinking status, thyroid function, liver function, and plasma lipid index. The threshold set for the Spearman’s correlation coefficient |R| and P-value was > 0.3 and < 0.05, respectively. Correlation networks between 10 clinical parameters and the top 40 abundant saliva microbial genera were visualized using Cytoscape (**Figure 4C**). Heatmap visualizing the correlation between clinical parameters and oral microbial genera were also displayed (**Figure S5A**).

The abundance levels of the XA group-enriched genera, including *fusobacterium*, *porphyromonas*, *campylobacter*, *dialister*, *prevotella*, *prevotella_7*, and *norank_f:Clostridiales_vadinBB60_group* was negatively correlated with USW/SWS. However, the abundance levels of the non-XA group-enriched genera, including *streptococcus*, were inversely related with USW/SWS (Student’s t-test, |R| > 0.3, P < 0.05, **Figures 4C, D** and **S5D, E**). In addition, the abundance of the XA group-enriched genera, including *fusobacterium*, *porphyromonas*, *treponema_2*, *campylobacter*, and *prevotella_2* was positively correlated with cumulative radioiodine dose. In contrast, cumulative radioiodine dose were negatively correlated with some of the non-XA group-enriched genera, including *streptococcus* (Student’s t-test, |R| > 0.3, P < 0.05)(**Figures 4C, D** and **S5B, C**). The abundance of oral *tannerella*, *catonella*, and *parvimonas* were positively correlated with OHI (Student’s t-test, |R| > 0.3, P < 0.05)(**Figures 4C** and **S5A**).

Redundancy analysis (RDA) also demonstrated the relationship between clinical parameters and oral microbial genera (**Figure 4E**). Concordant with PCoA, the oral microbiota was differentiated between the XA and non-XA groups. RDA showed that the cumulative radioiodine dose, saliva secretion (SWS, USW, and Scale), OHI score were positively correlated with the oral microbiota (permutation test P < 0.05), with SWS (permutation test, 43.42% variance explained, P = 0.001) and cumulative radioiodine dose (permutation test, 39.87% variance explained, P = 0.001) being the most correlated (**Figures 4E, F**). Age, Drinking, BMI, and smoking were not significantly related to the oral microbiota (**Figures 4E, F**).

### Functional Alterations in the Oral Microbiota of the Xerostomia Patients

**Figure 5:**
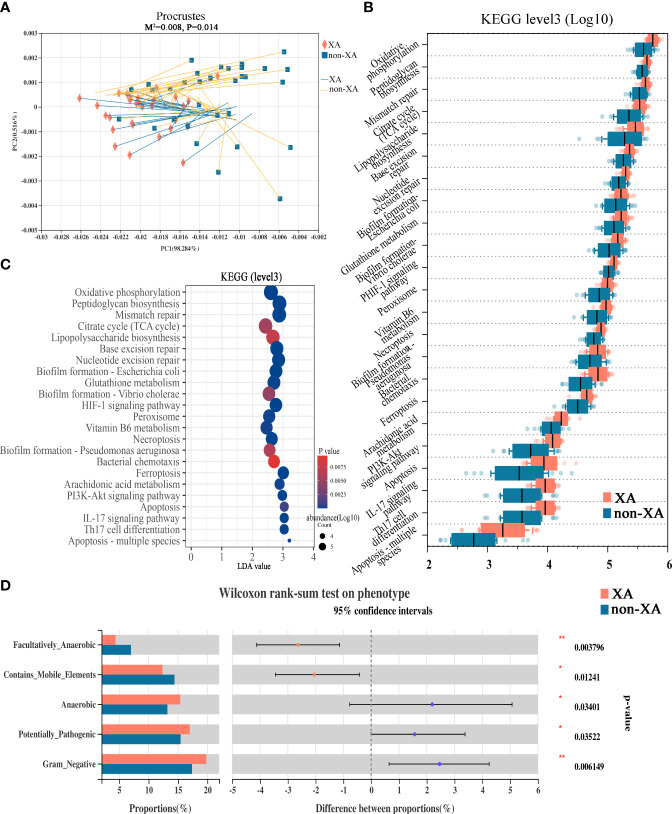
Functional alterations in the oral microbiota among XAs and non-XAs. **(A)** Procrustes analyses demonstrated a significant correlation between oral microbial composition and the potential function profile. The solid shape (square and cross) at one end of the line segment represented the sorting result of high-throughput sequencing data, the XA and non-XA groups were colored in orange and blue, respectively; The other end represented the ranking result of metabolic data, M^2^: the goodness-of-fit statistic of the ranking results in Platts analysis, used to evaluate the correlation between the ranking results, Monte Carlo P-value: indicates the value generated by Monte Carlo simulation P-value, used to test the significance of M^2^. **(B)** Twenty-three differential KEGG metabolic pathways were identified by PICRUSt2 (P < 0.05). **(C)** The bubble chart displayed 23 differentially abundant KEGG pathways by PICRUSt2. KEGG pathways with LDA values greater than two were retained. **(D)** BugBase phenotype prediction. XAs, xerostomia patients; non-XAs, patients without xerostomia. KEGG, Kyoto Encyclopedia of Genes and Genomes; LDA, linear discriminant analysis; PICRUSt2, Phylogenetic Investigation of Communities by Reconstruction of Unobserved States 2; * 0.01 < P < 0.05, ** 0.001 < P < 0.01.

Next, we assessed whether the drastic microbial community alteration would lead to the potential microbial functional consequence. There is a strong correlation between the oral microbial composition and microbial function profile evaluated by the Procrustes analysis (the global pattern)(M^2^ = 0.008, P = 0.014, **Figure 5A**). To analyze the differential functions of oral microbial communities between XA and non-XA groups, we performed PICRUSt2 analysis based on 16S rRNA sequencing data to predict the functional composition of microbial communities. Differentially enriched KEGG (level 3) metabolic pathways of oral microbial communities between the XA and non-XA groups were shown in **Figure 5B**. Among the 378 metabolic pathways tested, the abundance of 23 pathways was significantly different between the XA and non-XA groups with P value < 0.05 (**Figures 5B, C**). Inflammation-related pathways of Arachidonic acid metabolism, IL-17 signaling pathway, lipopolysaccharide (LPS) biosynthesis, and Peptidoglycan biosynthesis and antioxidant-related pathways of Glutathione metabolism, Vitamin B6 metabolism, and Oxidative phosphorylation were enriched in the XA group (P < 0.05, **Figures 5B, C**). Similarly, the metabolic pathways of HIF-1 signaling, PI3K-Akt, and Ferroptosis in the XA group were significantly up-regulated compared to the non-XA group (P < 0.05, **Figures 5B, C**). To further characterize the correlation of the different genera with differential KEGG metabolic pathways, Spearman’s correlation analysis results between 40 genera and 23 KEGG pathways were displayed using heatmap (Student’s t-test, |R| > 0.3, P < 0.05), as shown in **Figure S6**. *Porphyromonas*, *fusobacterium*, and *treponema_2* were positively correlated with inflammation and antioxidant-related metabolic pathways, while streptococcus is negatively correlated with the above pathways (**Figure S6**). In addition, we also predicted characteristic phenotypes of oral microbial communities by BugBase analysis. The results indicated that potentially_pathogenic, anaerobic, and gram_negative phenotypes were enriched in the XA group (P <0.05, **Figure 5D**). In contrast, mobile_element_containing, and facultatively_anaerobic were enriched in the non-XA group (P <0.05, **Figure 5D**). The above functional predictive analysis data reveal that dysregulation of inflammatory and antioxidant metabolic pathways and microbial phenotype transformation in patients with xerostomia may exacerbate the progression of xerostomia.

### Identification of Driver Microbial Taxa for Xerostomia

**Figure 6:**
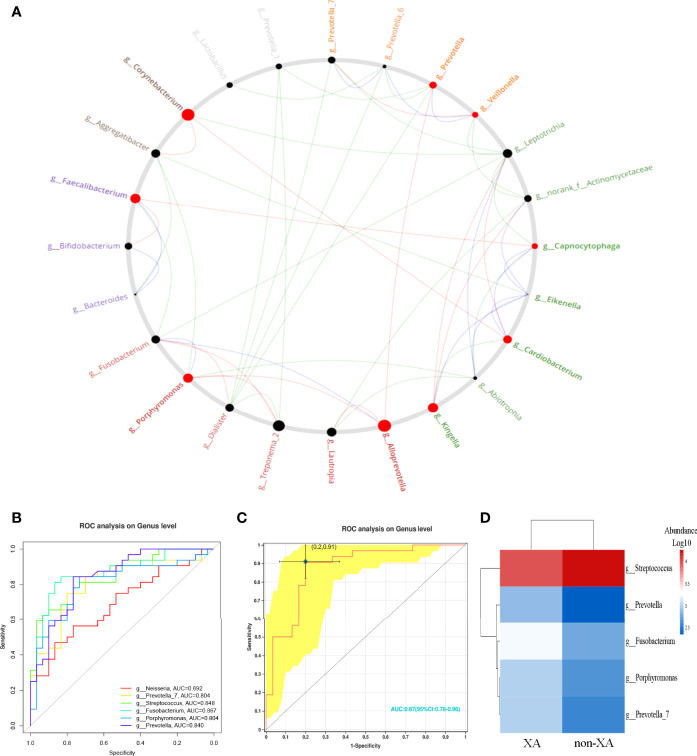
Identification of driver microbial taxa and prediction for xerostomia. **(A)** Identification of oral microbiota driver genera by performing NetShift analysis. The nodes marked in red represented the driver genera, and the size of the nodes represented the NESH score. Oral microbial genera showing pairwise positive correlations only in the non-XA group were shown with green line segments, genera showing positive correlations only in the XA group were shown with red line segments. In addition, blue line segments represented positive correlations between microbial genera among the non-XA and XA groups. **(B)** Six microbial genera with LDA values greater than four were selected as candidate features and five microbial genera (AUC > 0.7) **(C)** were further chosen as potential biomarkers. **(D)** Generated a heatmap based on the selected five predicted features. Hierarchical clustering confirmed that the predicted features of the two groups can be separated. The intensity of the squares represented the relative abundance of microbial genera. XA group, xerostomia group; non-XA group, without xerostomia group; LDA, linear discriminant analysis.

Microbial association networks for the XA and non-XA groups were created to identify statistically significant associations. The correlation network of oral microbial genera was constructed by the CCREPE software package. By performing the NetShift analysis between different oral microbial communities, *porphyromonas*, *corynebacterium*, *alloprevotella*, *kingella*, *faecalibacterium*, and *cardiobacterium* with higher NESH scores (bigger nodes) were identified as driver species, followed by *prevotella,veillonella*, and *capnocytophaga* with lower NESH scores (smaller nodes), as shown in **Figure 6A**. Among these identified driver genera, *porphyromonas* positively correlated with *aggregatibacter*, and *abiotrophia* in the non-XA group. However, *porphyromonas* was found to be positively associated with *treponema_2* and *alloprevotella* in the XA group. These results suggest that *porphyromonas* may be a community driver of xerostomia.

### Oral Microbiota Prediction of Xerostomia

According to the above results, we selected oral microbial genera with an LDA value greater than four as candidate features (**Figure 6B**) and finally screened six oral species (**Figure 6B**). Further, we limited the AUC value to 0.7, with five oral microbial genera as potential bacterial signatures (AUC ≥ 0.7). The model constructed based on the five bacterial signatures had robust diagnostic accuracy (AUC = 0.87, **Figure 6C**). The relative abundance of predictive biomarkers for xerostomia was applied to generate a heatmap (**Figure 6D**). The hierarchical clustering (Euclidean distance and complete linkage) showed a visible separation of predictive biomarkers between XAs and non-XAs (**Figure 6D**). The above results reveal that a diagnostic classifier based on oral microbiota can distinguish xerostomia patients from those without xerostomia after RAI.

## Discussion

Recent studies involving the interaction between the host and microbiota have revealed that oral microbial dysbiosis is closely associated with Sjogren’s syndrome (18, 25, 26). To date, the alterations in saliva microbiota related to the cumulative radioiodine dose/saliva secretion and its effect on the development of xerostomia in DTC patients after RAI have been seldom investigated in depth. Hence, our research aimed to assess and compare the oral microbial structure and related biological functions between XAs and non-XAs by applying 16S rRNA gene sequencing. In addition, correlation analysis between microbial genera and host clinical characteristics and identification of driver microbial taxa for xerostomia were also performed.

Higher richness in oral microbial composition was present in XAs compared to non-XAs in our study. This is consistent with a recent study demonstrating altered oral microbiota in patients with Sjögren’s syndrome (15). However, one study showed that the diversity of oral microbiota was reduced in Systemic lupus erythematosus patients compared to the healthy controls (25). In addition, the diversity of oral microbiota in patients with certain digestive system malignancies (such as colon cancer) was also significantly increased (27).

Firmicutes and Bacteroidetes were the dominant phyla in saliva microbiota, which is consistent with previous studies (28, 29). Meanwhile, we found that DTC patients with xerostomia after RAI had a higher F/B ratio of saliva microbiota compared with those without xerostomia. The abundance of *streptococcus* genus was decreased in XAs. This genus was known to benefit from establishing immune homeostasis and regulating host inflammation (30). In addition, the abundance of *neisseria*, *fusobacterium*, *veillonella*, *prevotella*, *porphyromonas*, *corynebacterium*, *capnocytophaga*, etc. was enriched in the saliva of XAs compared to those of the non-XA group. Some of them have long been considered as the primary oral pathogens of inducing excessive inflammation (31–34). *Prevotella* promotes inflammation by activating Toll-like receptor 2 and stimulating the immune response mediated by Th17 cells (35). Additionally, they are also producers of pathogenicity-related molecular patterns (PAMP). It is particularly worth noting that certain of these xerostomia-related species are indeed the most important causative agents associated with periodontitis, even including previously under-appreciated periodontal pathogens: such as *parvimonas*. *Bifidobacterium* and *lactobacillus* genera were well-known probiotics, although their relative abundance was increased in the XA group. Expectedly, *fusobacterium* was higher in the XA group, exhibiting an antagonist effect on beneficial bacteria (36). Thus, we infer that the imbalance of the ratio of anti-inflammatory bacteria and pro-inflammatory bacteria in the oral cavity after radioactive iodine treatment may aggravate salivitis and increase the eventual xerostomia incidence.

In our study, the RDA analysis indicated that the cumulative radioiodine dose of radioactive iodine and saliva secretion significantly contributed to variation in microbial profiles. *Fusobacterium*, *porphyromonas*, *treponema_2*, *campylobacter*, and *prevotella_2* were positively correlated with the levels of cumulative radioiodine dose. However, cumulative radioiodine dose levels were negatively associated with streptococcus. In nasopharyngeal cancer patients receiving radiotherapy, the radiation dose was positively correlated with *treponema* but negatively correlated with *prevotella*, *fusobacterium*, and *campylobacter (* 37). In addition, we found that *fusobacterium*, *porphyromonas*, *streptococcus* were associated with saliva secretion, which is different from the phenomenon previously observed in Sjogren’s syndrome patients (18). This result may be attributed to a different disease background.

The global pattern from Procrustes analysis displayed a good-fit correlation between the oral microbial community composition and microbial function in our study. Inflammation-related pathways [Arachidonic acid metabolism, IL-17 signaling pathway, lipopolysaccharide (LPS) biosynthesis], antioxidant-related pathway (Glutathione metabolism), and potentially_pathogenic were predicted by PICRUSt and BugBase analysis between the XA and non-XA groups. One possible explanation is that radioactive iodine therapy and subsequent reduction in saliva production may have shaped an abnormally metabolic microbiota. Arachidonic acid is the direct precursor of prostaglandin E2 (PGE2), prostaglandin (PGI2), thromboxane A2 (TXA2), leukotriene, and C4 (LTC4). The above products are produced through enzyme metabolism and cause different inflammation (38). Thromboxane and PGE2 in the saliva of patients with Sjogren’s syndrome increased significantly (39). Moreover, the study has shown that LPS can inhibit salivary secretion by increasing the production of prostaglandins (40). The LPS produced by *porphyromonas gingivalis* reduces mucin synthesis in salivary acinar cells, which is accompanied by acinar cell apoptosis and nitric oxide (NO) production (16). According to our study, *porphyromonas*, *fusobacterium*, and *treponema_2* were positively correlated with arachidonic acid metabolism and lipopolysaccharide (LPS) biosynthesis pathways. BugBase predictive analysis showed that the gram_negative phenotype was enriched in XAs, leading to high levels of LPS in the body (41). One study on the rat thyroid cell line FRTL-5 shows that the binding of gram-negative bacteria outer membrane LPS to Toll-like receptor 4 (TLR4) on thyroid cells activates the NF-κB signaling pathway, resulting in an increase in TSH-induced NIS mRNA expression in a dose-dependent manner upon LPS treatment (42). NIS plays an essential role in thyroid physiology and response to RAI through participating in iodide–uptake; overexpression of NIS may lead to an increase in iodide uptake. In addition, the salivary glands are more sensitive to the radiation effects of I-131 (43), which ultimately increases the incidence of radiation salivitis. Our data indicate that the dysregulation of inflammation-related and antioxidant-related pathways in DTC patients after RAI may contribute to the development of xerostomia.

The microbial community maintains its ecological balance through mutual symbiosis. Under certain disease conditions, a set of the key microbiota is likely to act as “drivers” (44). In our work, Netshift analysis showed that the microbial association network in the XA group was significantly different from that in the non-XA group. *Porphyromonas* was identified as a driver genus for xerostomia. Although those driving bacteria remained low relative abundance in this study. Just like the “keystone pathogen” hypothesis (44), we should not underestimate the roles they may play in xerostomia. Certain low-abundance bacteria, such as *porphyromonas*, can also collectively regulate activities including pathogen colonization, invasion, inflammation, etc. These activities will cause the overall oral microbiota to have more potent virulence (44–46).

Furthermore, it is also unclear if these observed changes in oral microbiota are a result of RAI, reduction of saliva secretion, or salivary gland inflammation. We infer that RAI may trigger early oral microbial dysbiosis and early salivary gland inflammatory response (radioactive salivitis) for the DTC patients. Continued inflammation will exert selective pressure to form highly dysbiotic disease aggravating communities, which may continue to amplify RAI-induced inflammation, thereby synergistically triggering a vicious cycle, resulting in aggravation of radioactive salivary gland inflammation and eventually forming xerostomia. Xerostomia can increase oral pathogens such as *fusobacterium*, reduced saliva secretion may further shape the microbiota, and oral microbiota can, in turn, worsen xerostomia. Thus, this study reveals the changes in the microbial composition of xerostomia after RAI in DTC patients and its relationship with clinical parameters for the first time. There are several limitations of this study. The major limitation is the insufficient sample size and sequencing technique. We implemented strict inclusion and exclusion criteria, which can lead to a dramatic reduction in the number of patients enrolled in the study. The functional prediction accuracy of 16S rRNA is relatively low. Shotgun generation sequencing metagenomics can precisely analyze the functions of different genes through functional annotation of gene sequences, which should be applied in future work. The subjective evaluation scales and standardized data collection may lead to potential observer bias. Nevertheless, the homogeneity of our study population is a vital quality criterion compared to other studies, which also included patients suffering from distant metastatic disease and taking drugs such as proton pump inhibitor (PPI) (26, 47). Drugs such as PPI can affect the oral microbiota composition (48). The results of this study are limited, and no major clinical consequences can be proven at this stage. The possible role of oral microbiota as “adjuvants for xerostomia” may become a starting point for radiologists and endocrinologists to clarify the interaction between RAI and microecology. So far, this correlation has not been fully evaluated. Our research provided new perspectives into the prevention, diagnosis, and treatment of xerostomia in DTC patients after RAI based on the oral microbiota. Meanwhile, exploring temporal follow-up of the changes in oral microbiota over time would be more convincing of the RAI-induced changes. In the future, the oral microbiota characteristics will be characterized at multiple time points before and after radioactive iodine treatment to evaluate the dynamic changes of the microbiota composition.

## Data Availability Statement

The original contributions presented in the study are publicly available. This data can be found here: NCBI, PRJNA784772.

## Ethics Statement

The studies involving human participants were reviewed and approved by the First Affiliated Hospital of Harbin Medical University. The patients/participants provided their written informed consent to participate in this study.

## Author Contributions

Guarantee all integrity associated with this study, YW, BL, FZ. Study concepts/study design or data acquisition or data analysis/interpretation, BL. Agree to be accountable for the content of the work, all authors. Collect the samples, YLi, YLu, JS, JF, LZ, HW, HC, WY, XG. Perform the bioinformatics and statistical analyses and interprets the data, SS, ZL, SW. Revise the manuscript for important content, YW, JZ. Manuscript drafting or manuscript revision for important intellectual content, all authors. Approval of the final version of the submitted manuscript, all authors. Agree to ensure any questions related to the work are appropriately resolved, all authors.

## Funding

This study is supported by Ningbo Digestive System Tumor Clinical Medical Research Center (2019A21003), China’s National Natural Science Foundation (NSFC81970466) and Natural Science Fundation of Fujian Province (No.2021J05143).

## Conflict of Interest

The authors declare that the research was conducted in the absence of any commercial or financial relationships that could be construed as a potential conflict of interest.

## Publisher’s Note

All claims expressed in this article are solely those of the authors and do not necessarily represent those of their affiliated organizations, or those of the publisher, the editors and the reviewers. Any product that may be evaluated in this article, or claim that may be made by its manufacturer, is not guaranteed or endorsed by the publisher.

## References

[1] Luster M Clarke SE Dietlein M Lassmann M Lind P Oyen WJ. European Association of Nuclear, Guidelines for Radioiodine Therapy of Differentiated Thyroid Cancer. Eur J Nucl Med Mol Imaging (2008) 35:1941–59. XXXM. doi: 10.1007/s00259-008-0883-1 18670773

[2] Jarzab B Handkiewicz-Junak D Wloch J. Juvenile Differentiated Thyroid Carcinoma and the Role of Radioiodine in its Treatment: A Qualitative Review. Endocr Relat Cancer (2005) 12:773–803. doi: 10.1677/erc.1.00880 16322322

[3] Malpani BL Samuel AM Ray S. Quantification of Salivary Gland Function in Thyroid Cancer Patients Treated With Radioiodine. Int J Radiat Oncol Biol Phys (1996) 35:535–40. doi: 10.1016/S0360-3016(96)80016-2 8655377

[4] Walter MA Turtschi CP Schindler C Minnig P Muller-Brand J Muller B. The Dental Safety Profile of High-Dose Radioiodine Therapy for Thyroid Cancer: Long-Term Results of a Longitudinal Cohort Study. J Nucl Med (2007) 48:1620–5. doi: 10.2967/jnumed.107.042192 17873131

[5] Stone HB Coleman CN Anscher MS McBride WH. Effects of Radiation on Normal Tissue: Consequences and Mechanisms. Lancet Oncol (2003) 4:529–36. doi: 10.1016/S1470-2045(03)01191-4 12965273

[6] Almeida JP Sanabria AE Lima EN Kowalski LP. Late Side Effects of Radioactive Iodine on Salivary Gland Function in Patients With Thyroid Cancer. Head Neck (2011) 33:686–90. doi: 10.1002/hed.21520 21484917

[7] Van Nostrand D Bandaru V Chennupati S Wexler J Kulkarni K Atkins F. Radiopharmacokinetics of Radioiodine in the Parotid Glands After the Administration of Lemon Juice. Thyroid (2010) 20:1113–9. doi: 10.1089/thy.2009.0429 20883172

[8] Klubo-Gwiezdzinska J Van Nostrand D Burman KD Vasko V Chia S Deng T. Salivary Gland Malignancy and Radioiodine Therapy for Thyroid Cancer. Thyroid (2010) 20:647–51. doi: 10.1089/thy.2009.0466 20470209

[9] Van Nostrand D Atkins F Bandaru VV Chennupati SP Moreau S Burman K. Salivary Gland Protection With Sialagogues: A Case Study. Thyroid (2009) 19:1005–8. doi: 10.1089/thy.2008.0381 19500022

[10] Mandel SJ Mandel L. Radioactive Iodine and the Salivary Glands. Thyroid (2003) 13:265–71. doi: 10.1089/105072503321582060 12729475

[11] Jentzen W Balschuweit D Schmitz J Freudenberg L Eising E Hilbel T. The Influence of Saliva Flow Stimulation on the Absorbed Radiation Dose to the Salivary Glands During Radioiodine Therapy of Thyroid Cancer Using 124I PET(/CT) Imaging. Eur J Nucl Med Mol Imaging (2010) 37:2298–306. doi: 10.1007/s00259-010-1532-z 20625723

[12] Dingle IF Mishoe AE Nguyen SA Overton LJ Gillespie MB. Salivary Morbidity and Quality of Life Following Radioactive Iodine for Well-Differentiated Thyroid Cancer. Otolaryngol Head Neck Surg (2013) 148:746–52. doi: 10.1177/0194599813479777 23462656

[13] Allweiss P Braunstein GD Katz A Waxman A. Sialadenitis Following I-131 Therapy for Thyroid Carcinoma: Concise Communication. J Nucl Med (1984) 25:755–8. 6737074

[14] Kim JW Kim JM Choi ME Kim SK Kim YM Choi JS. Does Salivary Function Decrease in Proportion to Radioiodine Dose? Laryngoscope (2020) 130:2173–8. doi: 10.1002/lary.28342 31765488

[15] Alam J Lee A Lee J Kwon DI Park HK Park JH. Dysbiotic Oral Microbiota and Infected Salivary Glands in Sjogren's Syndrome. PLoS One (2020) 15:e0230667. doi: 10.1371/journal.pone.0230667 32208441 PMC7092996

[16] Slomiany BL Slomiany A. Activation of Peroxisome Proliferator-Activated Receptor Gamma Impedes Porphyromonas Gingivalis Lipopolysaccharide Interference With Salivary Mucin Synthesis Through Phosphatidylinositol 3-Kinase/Erk Pathway. J Physiol Pharmacol (2003) 54:3–15. 12674215

[17] Tseng YC Yang HY Lin WT Chang CB Chien HC Wang HP. Salivary Dysbiosis in Sjogren's Syndrome and a Commensal-Mediated Immunomodulatory Effect of Salivary Gland Epithelial Cells. NPJ Biofilms Microbiomes (2021) 7:21. doi: 10.1038/s41522-021-00192-w 33707430 PMC7952914

[18] van der Meulen TA Harmsen HJM Bootsma H Liefers SC Vich Vila A Zhernakova A. Reduced Salivary Secretion Contributes More to Changes in the Oral Microbiome of Patients With Primary Sjogren's Syndrome Than Underlying Disease. Ann Rheum Dis (2018) 77:1542–4. doi: 10.1136/annrheumdis-2018-213026 29572289

[19] Thomson WM Chalmers JM Spencer AJ Williams SM. The Xerostomia Inventory: A Multi-Item Approach to Measuring Dry Mouth. Community Dent Health (1999) 16:12–7. 10697349

[20] Sreebny LM Valdini A. Xerostomia. Part I: Relationship to Other Oral Symptoms and Salivary Gland Hypofunction. Oral Surg Oral Med Oral Pathol (1988) 66:451–8. doi: 10.1016/0030-4220(88)90268-X 3186220

[21] Iakovou I Goulis DG Tsinaslanidou Z Giannoula E Katsikaki G Konstantinidis I. Effect of Recombinant Human Thyroid-Stimulating Hormone or Levothyroxine Withdrawal on Salivary Gland Dysfunction After Radioactive Iodine Administration for Thyroid Remnant Ablation. Head Neck (2016) 38 Suppl 1:E227–30. doi: 10.1002/hed.23974 25537365

[22] Lin B Zhao F Liu Y Wu X Feng J Jin X. Randomized Clinical Trial: Probiotics Alleviated Oral-Gut Microbiota Dysbiosis and Thyroid Hormone Withdrawal-Related Complications in Thyroid Cancer Patients Before Radioiodine Therapy Following Thyroidectomy. Front Endocrinol (Lausanne) (2022) 13:834674. doi: 10.3389/fendo.2022.834674 35350100 PMC8958007

[23] Wang Q Zhao L Han L Fu G Tuo X Ma S. The Differential Distribution of Bacteria Between Cancerous and Noncancerous Ovarian Tissues in Situ. J Ovarian Res (2020) 13:8. doi: 10.1186/s13048-019-0603-4 31954395 PMC6969417

[24] Kuntal BK Chandrakar P Sadhu S Mande SS. 'Netshift': A Methodology for Understanding 'Driver Microbes' From Healthy and Disease Microbiome Datasets. ISME J (2019) 13:442–54. doi: 10.1038/s41396-018-0291-x PMC6331612 30287886

[25] van der Meulen TA Harmsen HJM Vila AV Kurilshikov A Liefers SC Zhernakova A. Shared Gut, But Distinct Oral Microbiota Composition in Primary Sjogren's Syndrome and Systemic Lupus Erythematosus. J Autoimmun (2019) 97:77–87. doi: 10.1016/j.jaut.2018.10.009 30416033

[26] van der Meulen TA Harmsen HJM Bootsma H Liefers SC Vich Vila A Zhernakova A. Dysbiosis of the Buccal Mucosa Microbiome in Primary Sjogren's Syndrome Patients. Rheumatol (Oxford) (2018) 57:2225–34. doi: 10.1093/rheumatology/key215 30060225

[27] Wang Y Zhang Y Qian Y Xie YH Jiang SS Kang ZR. Alterations in the Oral and Gut Microbiome of Colorectal Cancer Patients and Association With Host Clinical Factors. Int J Cancer (2021). doi: 10.1002/ijc.33596 33844851

[28] Aas JA Paster BJ Stokes LN Olsen I Dewhirst FE. Defining the Normal Bacterial Flora of the Oral Cavity. J Clin Microbiol (2005) 43:5721–32. doi: 10.1128/JCM.43.11.5721-5732.2005 PMC1287824 16272510

[29] Bik EM Long CD Armitage GC Loomer P Emerson J Mongodin EF. Bacterial Diversity in the Oral Cavity of 10 Healthy Individuals. ISME J (2010) 4:962–74. doi: 10.1038/ismej.2010.30 PMC2941673 20336157

[30] Kaci G Goudercourt D Dennin V Pot B Dore J Ehrlich SD. Anti-Inflammatory Properties of Streptococcus Salivarius, a Commensal Bacterium of the Oral Cavity and Digestive Tract. Appl Environ Microbiol (2014) 80:928–34. doi: 10.1128/AEM.03133-13 PMC3911234 24271166

[31] Farrow B Evers BM. Inflammation and the Development of Pancreatic Cancer. Surg Oncol (2002) 10:153–69. doi: 10.1016/S0960-7404(02)00015-4 12020670

[32] Mueller MM. Inflammation in Epithelial Skin Tumours: Old Stories and New Ideas. Eur J Cancer (2006) 42:735–44. doi: 10.1016/j.ejca.2006.01.014 16527478

[33] Feller L Altini M Lemmer J. Inflammation in the Context of Oral Cancer. Oral Oncol (2013) 49:887–92. doi: 10.1016/j.oraloncology.2013.07.003 23910564

[34] Atarashi K Suda W Luo C Kawaguchi T Motoo I Narushima S. Ectopic Colonization of Oral Bacteria in the Intestine Drives TH1 Cell Induction and Inflammation. Science (2017) 358:359–65. doi: 10.1126/science.aan4526 PMC5682622 29051379

[35] Larsen JM. The Immune Response to Prevotella Bacteria in Chronic Inflammatory Disease. Immunology (2017) 151:363–74. doi: 10.1111/imm.12760 PMC5506432 28542929

[36] Guo S Li L Xu B Li M Zeng Q Xiao H. And Novel Fecal Biomarker for Colorectal Cancer: Ratio of Fusobacterium Nucleatum to Probiotics Populations, Based on Their Antagonistic Effect. Clin Chem (2018) 64:1327–37. doi: 10.1373/clinchem.2018.289728 29914865

[37] Hou J Zheng H Li P Liu H Zhou H Yang X. Distinct Shifts in the Oral Microbiota are Associated With the Progression and Aggravation of Mucositis During Radiotherapy. Radiother Oncol (2018) 129:44–51. doi: 10.1016/j.radonc.2018.04.023 29735410

[38] Wang T Fu X Chen Q Patra JK Wang D Wang Z. Arachidonic Acid Metabolism and Kidney Inflammation. Int J Mol Sci 20 (2019) 20(15):3683. doi: 10.3390/ijms20153683 PMC6695795 31357612

[39] Tishler M Yaron I Raz A Meyer FA Yaron M. Salivary Eicosanoid Concentration in Patients With Sjogren's Syndrome. Ann Rheum Dis (1996) 55:202–4. doi: 10.1136/ard.55.3.202 PMC1010131 8712887

[40] Lomniczi A Mohn C Faletti A Franchi A McCann SM Rettori V. Inhibition of Salivary Secretion by Lipopolysaccharide: Possible Role of Prostaglandins. Am J Physiol Endocrinol Metab (2001) 281:E405–11. doi: 10.1152/ajpendo.2001.281.2.E405 11440919

[41] Ruhal R Kataria R. Biofilm Patterns in Gram-Positive and Gram-Negative Bacteria. Microbiol Res (2021) 251:126829. doi: 10.1016/j.micres.2021.126829 34332222

[42] Nicola JP Nazar M Mascanfroni ID Pellizas CG Masini-Repiso AM. NF-kappaB P65 Subunit Mediates Lipopolysaccharide-Induced Na(+)/I(-) Symporter Gene Expression by Involving Functional Interaction With the Paired Domain Transcription Factor Pax8. Mol Endocrinol (2010) 24:1846–62. doi: 10.1210/me.2010-0102 PMC5417406 20667985

[43] Jeong SY Kim HW Lee SW Ahn BC Lee J. Salivary Gland Function 5 Years After Radioactive Iodine Ablation in Patients With Differentiated Thyroid Cancer: Direct Comparison of Pre- and Postablation Scintigraphies and Their Relation to Xerostomia Symptoms. Thyroid (2013) 23:609–16. doi: 10.1089/thy.2012.0106 PMC3643252 23153322

[44] Hajishengallis G Darveau RP Curtis MA. The Keystone-Pathogen Hypothesis. Nat Rev Microbiol (2012) 10:717–25. doi: 10.1038/nrmicro2873 PMC3498498 22941505

[45] Hajishengallis G. Immunomicrobial Pathogenesis of Periodontitis: Keystones, Pathobionts, and Host Response. Trends Immunol (2014) 35:3–11. doi: 10.1016/j.it.2013.09.001 24269668 PMC3947349

[46] Hajishengallis G. The Inflammophilic Character of the Periodontitis-Associated Microbiota. Mol Oral Microbiol (2014) 29:248–57. doi: 10.1111/omi.12065 PMC4232466 24976068

[47] Selvakumar T Nies M Klein Hesselink MS Brouwers AH van der Horst-Schrivers ANA Klein Hesselink EN. Long-Term Effects of Radioiodine Treatment on Salivary Gland Function in Adult Survivors of Pediatric Differentiated Thyroid Carcinoma. J Nucl Med (2018) 30. doi: 10.2967/jnumed.118.212449 30504138

[48] Mishiro T Oka K Kuroki Y Takahashi M Tatsumi K Saitoh T. Oral Microbiome Alterations of Healthy Volunteers With Proton Pump Inhibitor. J Gastroenterol Hepatol (2018) 33:1059–66. doi: 10.1111/jgh.14040 29105152

