# Supplementary material for: Alterations in Oral Microbiota of Differentiated Thyroid Carcinoma Patients With Xerostomia After Radioiodine Therapy
Source: Front Endocrinol (Lausanne). 2022 Aug 26;13:895970. doi: 10.3389/fendo.2022.895970 (PMC9459331; doi:10.3389/fendo.2022.895970)
Supplement: Supplementary file 1 [file DataSheet_1.docx]

Supplementary Material

# Supplementary Data

## Exclusion criteria

Pregnancy; lactation; severe periodontal disease; cardiovascular and metabolic diseases, such as hypertension, diabetes mellitus, and BMI > 27; use corticosteroids at the time of RAI treatment; recent (< 3 months prior) use of antibiotics, laxatives, proton pump inhibitors, insulin sensitizers, or traditional Chinese medicine and other drugs that may affect salivary gland function; known history of symptoms related to salivary dysfunction (dry mouth, salivary gland pain, and swelling) before RAI treatment; known history of disease with an autoimmune component, such as Sjogren's syndrome, systemic lupus erythematosus, multiple sclerosis, rheumatoid arthritis, irritable bowel syndrome, or IBD; and history of malignancy or any gastrointestinal tract surgery (e.g., gastrointestinal surgery, cholecystectomy or appendectomy).

## Plasma and oral sample collection

All participants were examined in the morning after an overnight fast (≥ 8 h). Peripheral plasma (15 mL) was collected from all subjects and stored in corresponding plasma collection tubes at 4 °C for the detection of thyroid function indicators (free triiodothyronine, [fT3], free thyroxine, [fT4], and thyrotropin, [TSH], thyroglobulin, [Tg]) examinations, liver function (alanine aminotransferase [ALT], aspartate transaminase [AST], album [ALB], total protein [TP], globulin [GLB), total bilirubin [TBIL], direct bilirubin [DBIL], indirect bilirubin [IBIL], gamma-glutamyl transpeptidase [GGT], alkaline phosphatase [AKP], plasma lipid (total cholesterol [CHOL], triacylglycerol [TG], low-density lipoprotein [LDL], apolipoprotein A [ApoA], high-density lipoprotein [HDL], very low density lipoprotein [VLDL], apolipoprotein B [ApoB], lipoprotein(a) [Lpa]). Participants were prohibited from eating, drinking liquids other than water, and doing oral hygiene activities at least one h before sampling. From each patient, salivary was collected for subsequent 16S rRNA gene analysis. All samples taken were frozen immediately at -20^。^ C and then stored at -80^。^C until analysis.

## Analysis of clinical parameters

Serum fT3, fT4, TSH, and Tg levels were measured by a chemiluminescent immunoassay (Abbott Diagnostics, Tokyo, Japan). Liver function and plasma lipid indexes were assessed using an automated biochemistry analyzer (Beckman Coulter, California, USA) and auxiliary reagents.

# Supplementary Figures


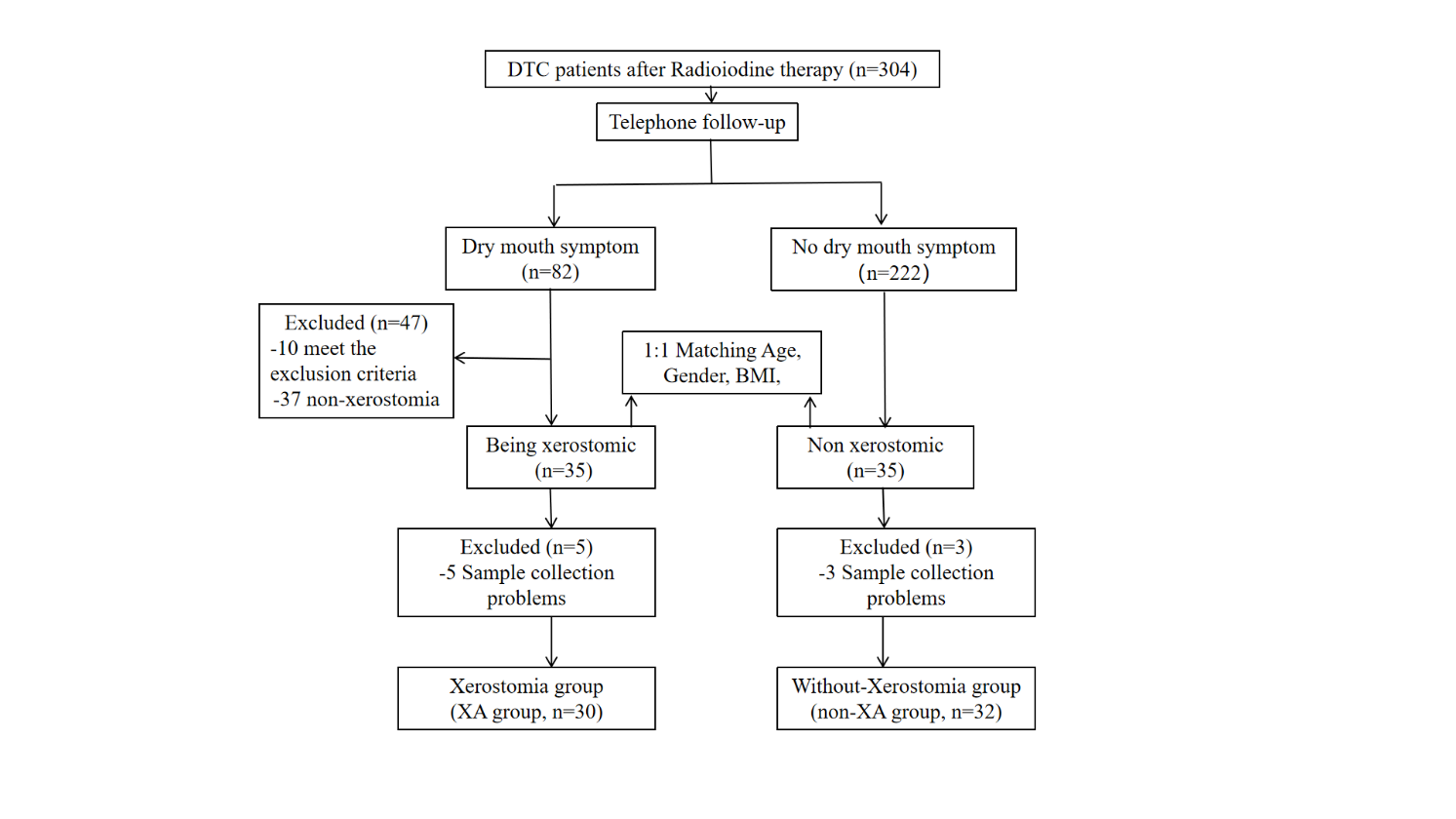


**Supplementary Figure 1.** Flow chart of patient recruitment


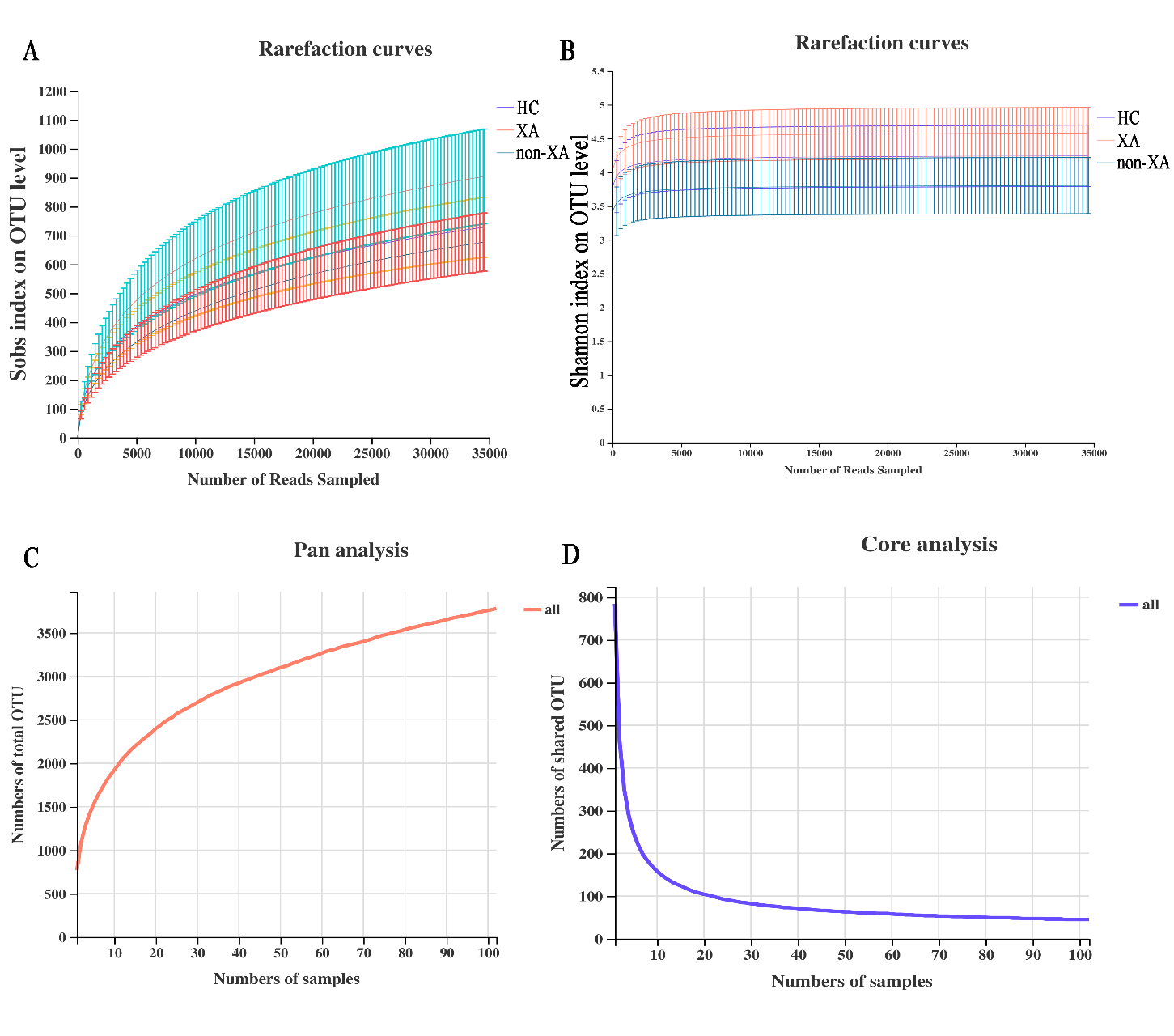


**Supplementary Figure 2.** Rarefaction analysis between the number of Reads Sampled and Sobs (A) and Shannon (B) index on OTU level. As the number of Reads Sampled increased, the number of OTUs approached saturation in XAs (n=30), non-XAs (n=32), and HCs (n=40). Compared with the non-XAs and HCs, the Sobs and Shannon index on OTU level in XAs was increased. (C-D) Pan/core species curves also demonstrated that the sequencing sample size was adequate. XAs, Xerostomia patients; non-XAs, without Xerostomia patients; HCs, healthy controls.


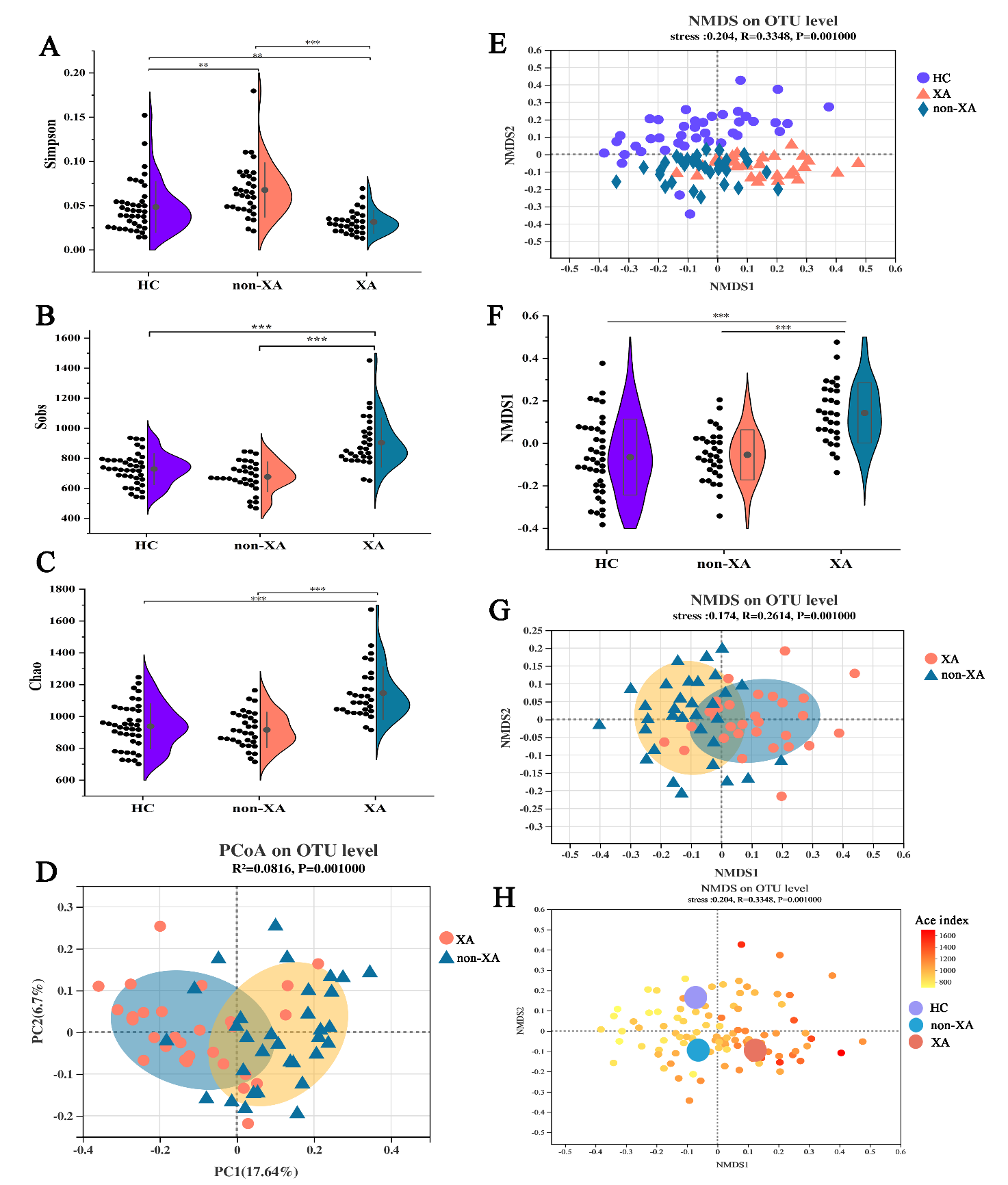


**Supplementary Figure 3.** The alpha diversity index of Simpson (A), Sobs (B), and Chao index (C) are significantly different among the three groups. (D) The principal coordinate analysis (PCoA) based on binary-chord distances at the OTU level showed that the oral microbial communities in the XAs are significantly different from non-XAs. (E-F) Non-metric multidimensional scaling (NMDS) based on unweighted-unifrac distances at OTU level for oral microbiota between XA, non-XA, and HC groups. (G) Non-metric multidimensional scaling (NMDS) based on unweighted-unifrac distances at OTU level for oral microbiota between XA and non-XA group. (H) NMDS as in (E), colored according to ace index. XAs, xerostomia patients; non-XAs, without xerostomia patients; HCs, healthy controls; OTUs, operational taxonomic units. * 0.01 < P < 0.05，** 0.001 < P < 0.01，*** P < 0.001.


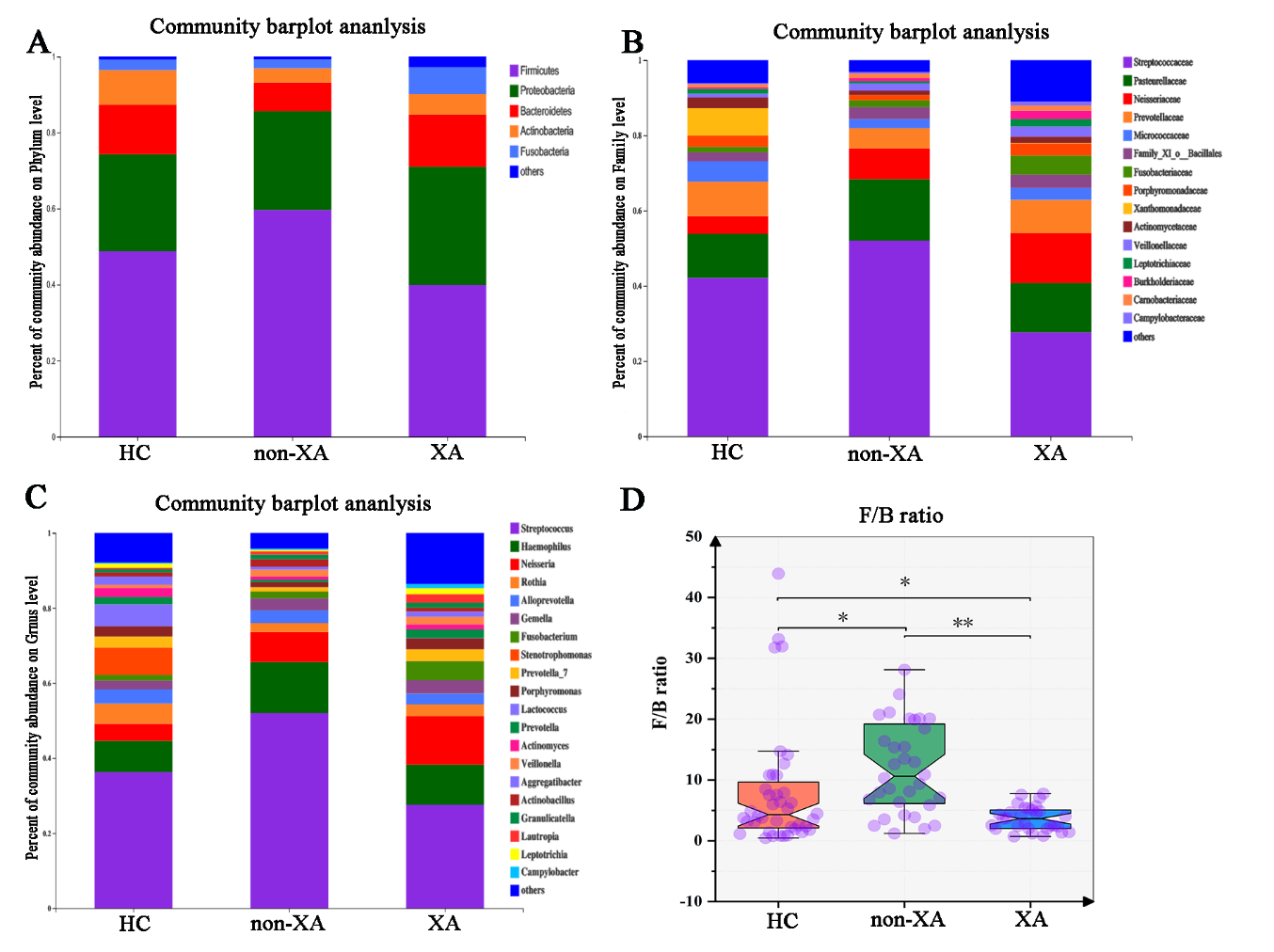


**Supplementary Figure 4.** Oral microbiota composition and F/B ratio. The relative abundances of the oral bacterial phylum (A), family (B), and genera (C) clustered into different groups were determined, revealing that the microbiota compositions differed significantly. (D) Box plot showing the F/B index of the oral microbiota among the XA, non-XA, and HC groups. XAs, xerostomia patients; non-XAs, without xerostomia patients; HCs, healthy controls; F/B ratio, Firmicutes/Bacteroidetes ratio; * 0.01 < P < 0.05，** 0.001 < P < 0.01，*** P < 0.001.


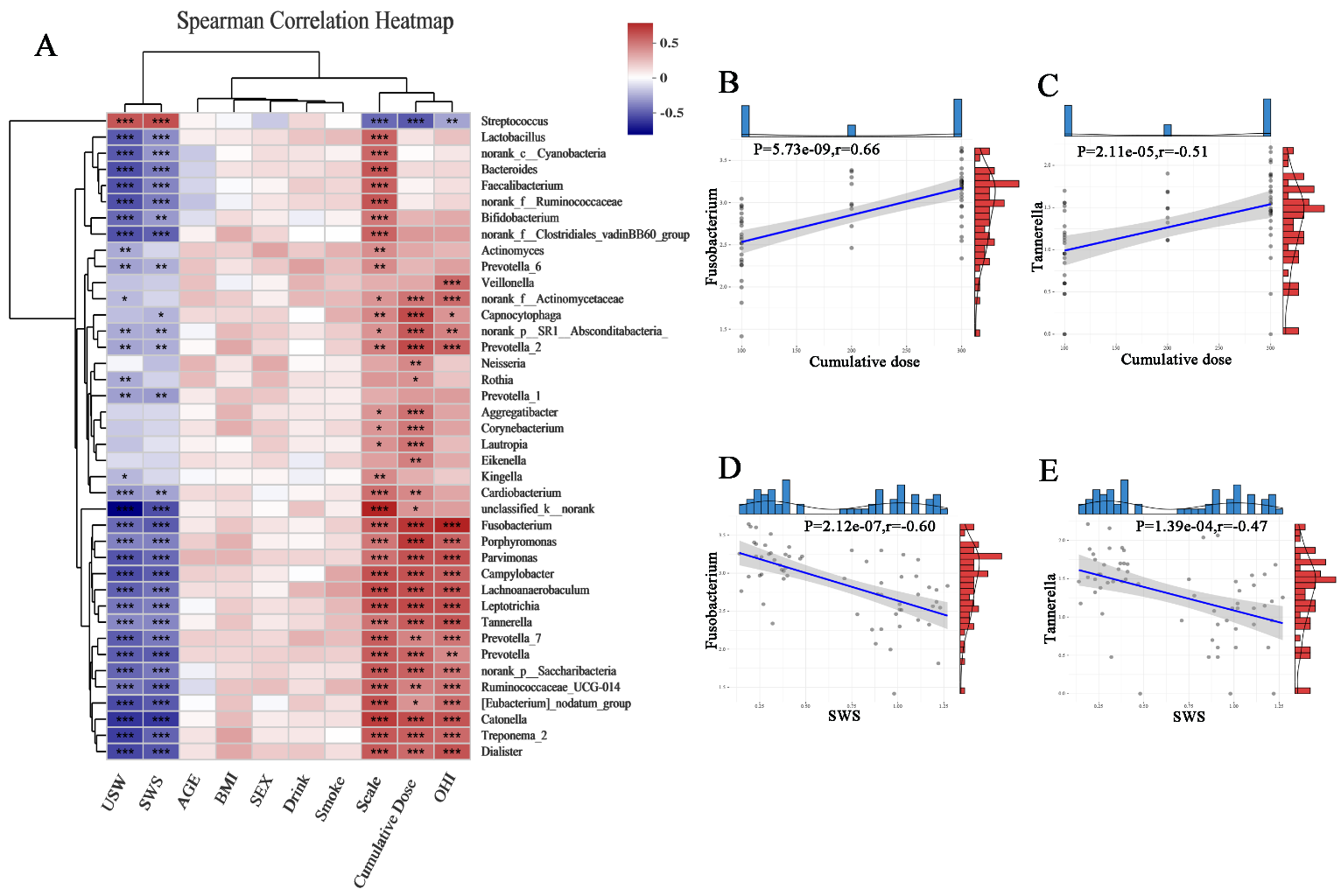


**Supplementary Figure 5.** Association of oral microbiota with clinical parameters. (A) The relationships among ten clinical indicators and 40 differentially abundant genera (Figure 2B-C) were estimated using Spearman correlation analysis. The results (B-E) Linear correlation analysis demonstrated a significant correlation between the relative abundance of fusobacterium, tannerella, and cumulative dose, SSW in the XAs and non-XAs; * 0.01 < P < 0.05，** 0.001 < P < 0.01，*** P < 0.001.


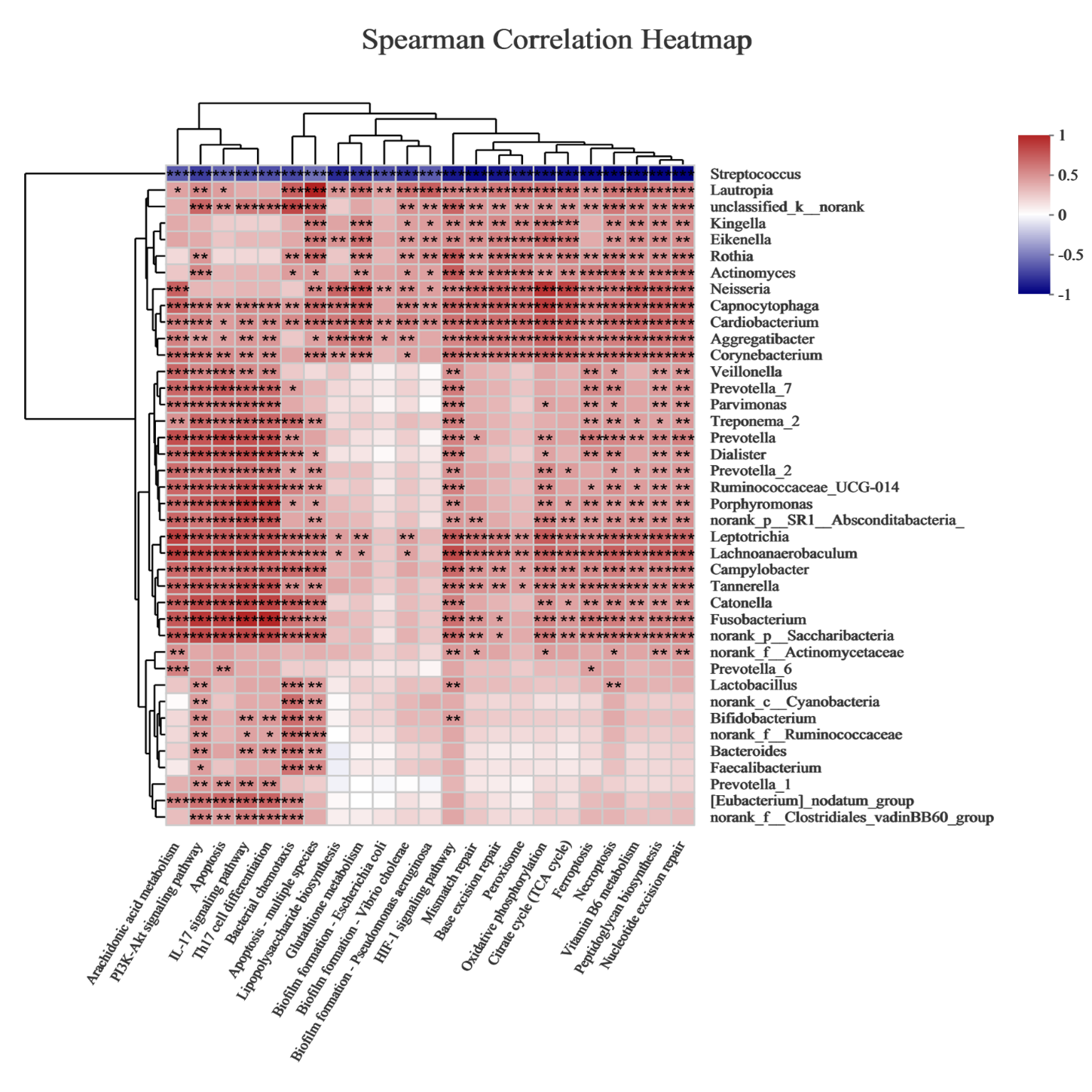


**Supplementary Figure 6.** The relationships among 40 differentially abundant genera (Figure 2B-C) and 23 KEGG metabolic pathways (Figure 5B-C) were estimated using Spearman correlation analysis. Color intensity represents the magnitude of correlations. Red, positive correlations; blue, negative correlations. Spearman correlation coefficient values below −0.3 (negative correlation) are indicated as red edges, and coefficient values above 0.3 (positive correlation) are indicated as green edges. * 0.01 < P < 0.05，** 0.001 < P < 0.01，*** P < 0.001.
